# A Whole-Genome Microarray Study of *Arabidopsis thaliana* Semisolid Callus Cultures Exposed to Microgravity and Nonmicrogravity Related Spaceflight Conditions for 5 Days on Board of Shenzhou 8

**DOI:** 10.1155/2015/547495

**Published:** 2015-01-13

**Authors:** Svenja Fengler, Ina Spirer, Maren Neef, Margret Ecke, Kay Nieselt, Rüdiger Hampp

**Affiliations:** ^1^Physiological Ecology of Plants, University of Tübingen, Auf der Morgenstelle 1, 72076 Tübingen, Germany; ^2^Center for Bioinformatics, University of Tübingen, Sand 14, 72076 Tübingen, Germany

## Abstract

The Simbox mission was the first joint space project between Germany and China in November 2011. Eleven-day-old *Arabidopsis thaliana* wild type semisolid callus cultures were integrated into fully automated plant cultivation containers and exposed to spaceflight conditions within the Simbox hardware on board of the spacecraft Shenzhou 8. The related ground experiment was conducted under similar conditions. The use of an in-flight centrifuge provided a 1 g gravitational field in space. The cells were metabolically quenched after 5 days via RNA*later* injection. The impact on the *Arabidopsis* transcriptome was investigated by means of whole-genome gene expression analysis. The results show a major impact of nonmicrogravity related spaceflight conditions. Genes that were significantly altered in transcript abundance are mainly involved in protein phosphorylation and MAPK cascade-related signaling processes, as well as in the cellular defense and stress responses. In contrast to short-term effects of microgravity (seconds, minutes), this mission identified only minor changes after 5 days of microgravity. These concerned genes coding for proteins involved in the plastid-associated translation machinery, mitochondrial electron transport, and energy production.

## 1. Introduction

Gravitation biology is a field of research which has made considerable progress within the last years, involving prokaryotes, fungi, plants, and animals. Plants are especially interesting, because, as sessile organisms, they possess high versatility in responding to environmental challenges and abiotic as well as biotic ones. In order to investigate responses to altered gravitation, a large range of methods is available that allows for modification of the Earth's gravitational field. These involve centrifugation (hypergravity), clinorotation, magnetic levitation, and random positioning (simulated microgravity), or parabolic flights of planes and sounding rockets, as well as satellites and spacecrafts (deliver microgravity). Experiments with plants show that not only tissues and organelles [1, 2] but also single-cell systems like characean rhizoids [3–7] as well as spores (*Ceratopteris richardii*, [8, 9]) and protoplasts [10–12] or homogeneous cell cultures (*Arabidopsis thaliana*) exhibit gravisensitivity [2, 13–16]. Experimental approaches that analyze the response to altered gravitation such as transcriptomics, proteomics, and metabolomics dominate recently. First molecular approaches were using transcriptomics, that is, the search for genes which change their expression under altered gravitation. In plants, like in other organisms, the improvement of gene expression quantification technologies, together with growing databases, supports this development considerably. To date, databases are available that exhibit plant datasets representing their response to diverse experimental stimuli [17–20]. They show that external signals are translated into biochemical ones, resulting in molecular signaling cascades which eventually result in a life-sustaining adaptation process.

For* Arabidopsis* (*Arabidopsis thaliana*) cell suspension cultures, the response to short-term microgravity was investigated intensely in our group by means of parabolic flights [21]. A combination of transcriptomics with phosphoproteomics showed that changes in gene expression and protein modification occur within seconds. The investigation of effects caused by longer-lasting microgravity depends on much scarcer availability of respective flight opportunities. However, data on cellular and molecular long-term responses of plants such as Brassicaceae (*Arabidopsis*), Fabaceae, and Poaceae has recently been published [2, 15, 22–31]. With regard to long-term experiments on gene expression, there are conflicting reports. Stutte et al. [30], for example, could not observe differentially expressed genes (DEGs) above a 2-fold cut-off in 24-day-old wheat leaves after a 21-day-space mission. In contrast, Paul et al. [15, 24] detected many DEGs in nearly 20-day-old* Arabidopsis* callus cultures and 18-day-old seedlings after a nearly 13-day-space mission. Furthermore, the set of altered genes detected in whole seedlings was different from that in callus cultures [15]. Thereby, the spaceflight-mediated upregulated expression of heat shock proteins appeared to be an age-independent cell culture specific response [15, 16]. Within the so-called TROPI-2 experiment, only 24 genes were altered in their abundance in* Arabidopsis* seedlings [2], due to possible microgravity effects after 4 days. In addition, these authors reported differences between the 1 g ground sample and the 1 g in-flight controls, with over 200 DEGs [2]. Also Zhang et al. [32] observed a greater difference between flight and ground samples with respect to 1 g in-flight conditions. These observations indicate that the differing results could be related to the organisms investigated, the time of exposure, hardware, experimental parameters, and set-up.

In this study, we report on results of a spaceflight experiment. This experiment was part of the Simbox (Science in Microgravity Box) mission, a joint project between the space agencies from Germany (Deutsches Zentrum für Luft- und Raumfahrt e. V.) and China (China Manned Space Engineering) in November 2011. As one out of 17 biological experiments, semisolid callus cultures of* Arabidopsis* were exposed to a 17-day spaceflight on board of the Chinese spacecraft Shenzhou 8. Due to reduced viability after longer periods of exposure within the flight hardware, the callus cultures were metabolically quenched after 5 days in space. Results of a whole-genome microarray screening (*μ*g exposed samples, 1 g in-flight samples kept in a reference centrifuge, and 1 g ground samples) revealed major differences between both 1 g controls but a minor impact of microgravity.

## 2. Material and Methods

### 2.1. Experiment-Specific Hardware (HW)

**Figure 1:**
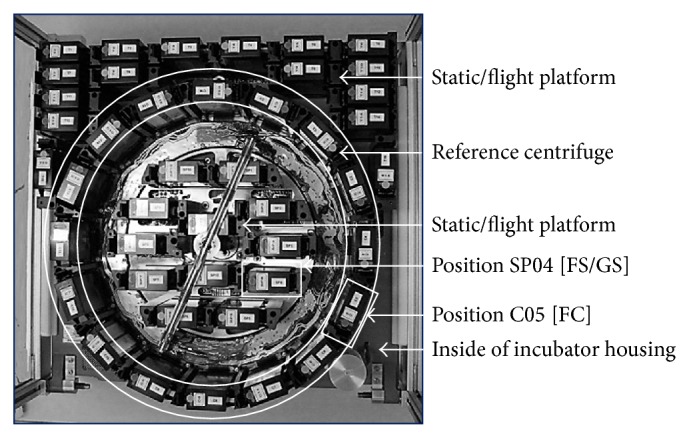
Photograph of the inside of the Simbox incubator used within the flight/ground experiment (housing removed). The rotor of the reference centrifuge (position C05 for sample group FC) is indicated by a circle. The static experimental platform is in the middle and outside of the centrifuge rotor (position SP04 for sample group FS within the flight experiment and GS within the ground experiment, resp.) (photograph: DLR/Astrium).

**Figure 2:**
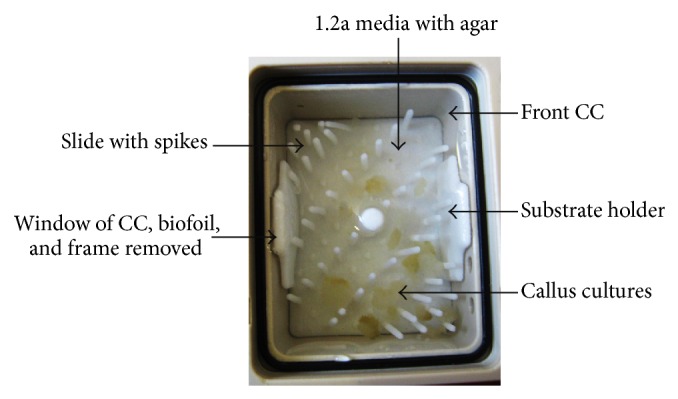
Photograph of the inside of one culture chamber (CC) (experiment container (EC), window, biofoil, and frame removed). The semisolid callus cultures were positioned on substrate holders (slides) with plastic spikes on 1.2a agar containing culture media.

**Figure 3:**
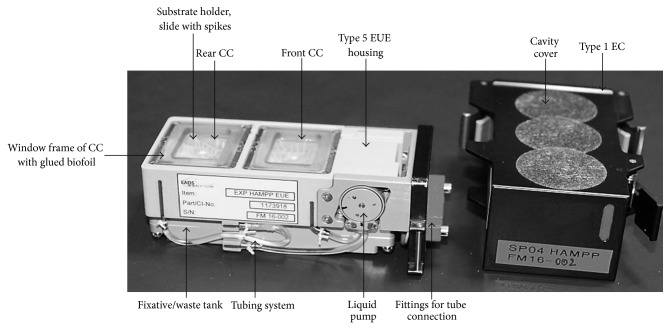
Photograph of the fully automated plant cultivation unit, type V EUE (left side), and EC removed (right side) (photograph: DLR/Astrium).

The Simbox was a modification of the Biobox-6 [33, 34] which was developed for unmanned recoverable capsules and space shuttle missions. Development and production were carried out by Astrium/EADS, Friedrichshafen, Germany [35]. This incubator (size of 461 × 551 × 273 mm, internal volume of 34 L, max. power consumption of 130 W, and empty mass of 17 kg) served as carrier for an experiment/static platform with an integrated centrifuge rotor (provides 1 g in-flight control). The Simbox incubator (Figure 1) enabled sample cultivation at 22–24°C (nominal temperature range) and 30–40% humidity throughout the mission. A duplicate model of the Simbox was constructed for the ground experiment. Our biological approach (experiment number 16) was realized by means of three fully automated type V Experiment Unit Envelopes (EUE, plant cultivation unit, without illumination). EUEs consisted of support housing made of polyetherketone with two culture chambers each (front and rear CC, 31.7 × 24 × 14.3 mm ± 0.15 mm). Our biological material was positioned on substrate holders (slides) with plastic spikes (Figure 2). The latter were needed to keep the cultures in place. In order to allow gas exchange, the CCs were sealed with a biofoil made of polysulfone (Tecason S Polysulfone, Ensinger Inc., Washington-Pennsylvania, USA). In addition, a peristaltic pump (flow rate of ≥2.43 mL/min) was used to connect the CC to a fixative/waste unit (volume 20.3 mL ± 0.5 mL). EUEs were accommodated inside type I Experiment Containers (ECs) (Figure 3). Via sensors, parameters such as temperature, humidity, CO_2_, and O_2_ content as well as activation of the pump system were recorded and transmitted.

### 2.2. Cell Cultures

Sterile cuttings (about 50 mm long) of stems of wild type* Arabidopsis thaliana* (cv. Columbia Col-0) plants were used for callus formation on 1.2a media [36] containing 1% agar (Sigma-Aldrich, Germany). Calli were transferred to 500 mL Erlenmeyer flasks with 200 mL liquid 1.2a medium and cultivated under sterile conditions at 23°C in the dark on a rotary shaker (130 rpm, Infors, Bottmingen, Switzerland), as described previously [14]. New medium was added every week to the resulting cell suspension. Eight months before the Simbox mission, an aliquot of this culture (3 g) was spread on 6 cm Petri dishes (Greiner Bio-One, Frickenhausen, Germany) containing agar and 1.2a medium. Cell cultures were mailed to the Institute of Physiology and Ecology, Shanghai (Laboratory of Prof. Zheng), and the cultivation continued (as liquid suspension) as described above. These suspension cultures were transferred to the PITC (Payload Integration and Test Center, Beijing, China). The cultivation was then continued on agar plates (see above) and, finally these semisolid calli were brought to the launch site (Jiuquan Satellite Launch Center, Jiuquan, China) by plane.

### 2.3. Preparation of Final Experiment Configuration

**Figure 4:**
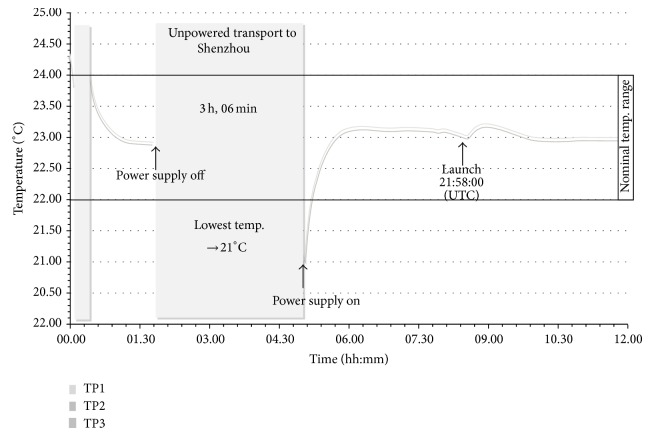
Temperature profile as recorded by 3 temperature sensors (TP1-3) attached to the Simbox incubator during integration of ECs into the incubator, transport to Shenzhou, and launch (data: Astrium).

One day before the launch, 11-day-old semisolid callus cultures were transferred into the CCs with 2 mL agar containing medium (Figures 2 and 3). Two ECs were used for the spaceflight (flight models: FM 16001 and FM 16002) and one for the ground experiment (FM 16003), respectively. One of the two ECs was contained in the centrifuge rotor, and the other one was fixed at the experiment/static platform (flight platform), respectively (Figure 1). Metabolic quenching of the samples was by the injection of RNA*later* (Ambion, Life Technologies, Darmstadt, Germany). This reagent is also used to stabilize nucleic acids. Twenty mL of this fixative was filled into the fixative/waste unit attached to the bottom of the EC. Between handover and integration into the Simbox flight/ground incubator, the ECs were stored at nominal laboratory temperature conditions (22–24°C). The Simbox incubator was unpowered for about 3 hours during transport to the spacecraft. During this time, the lowest temperature was 21°C (Figure 4).

### 2.4. The Experiment in Orbit

**Figure 5:**
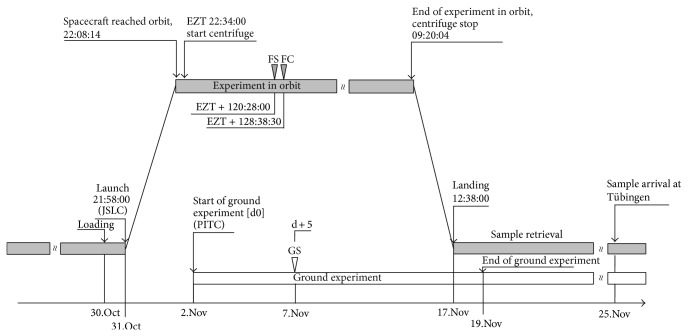
Precise mission timeline of the experiment in orbit (grey) and related ground experiment (white). Universal time coordinated (UTC), time units are given in hours:minutes: seconds, experimental zero time (EZT). Arrowheads (∇) indicate sample fixation time points of sample groups FS, FC, and GS, respectively.

**Figure 6:**
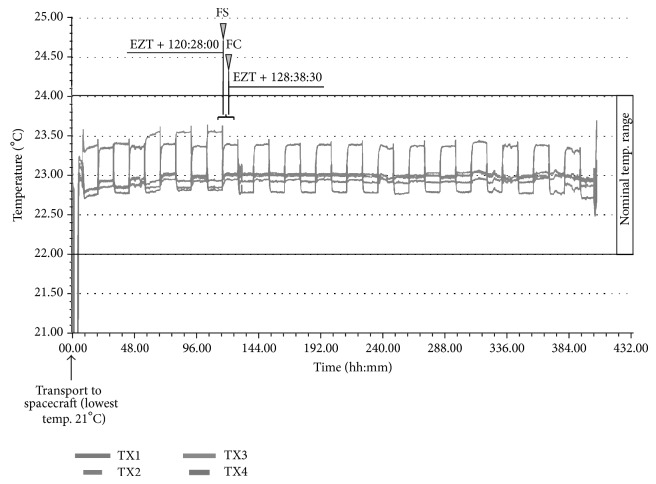
Temperature profile as recorded by 4 temperature sensors (TX1-4) attached to the Simbox incubator during the whole Simbox mission (data: Astrium). Sample fixation time points for the spaceflight samples (FS and FC) are indicated by arrowheads (grey triangle).

The Simbox was launched on board of the unmanned spacecraft Shenzhou 8 on October 31, 2011, at 21:58 UTC (universal time coordinated) with a Long March 2F rocket from the cosmodrome in JSLC. The precise mission timings including sample fixation time points are illustrated in Figure 5 (for a gravity-level profile, see Supplementary Material S1 available online at http://dx.doi.org/10.1155/2014/547495). Experiment zero time (EZT) was set when the spacecraft reached the orbit. At EZT, the centrifuge was activated to run with 74.40 rpm. Within the spacecraft, the oxygen partial pressure ranged from 18.04 to 27.32 kPa, and the carbon dioxide partial pressure was between −0.03 and 0.46 kPa. Radiation measurements yielded a total dose of 5.93 to 8.1 mSv and an equivalent dose of 0.37 to 0.51 mSv/d near the Simbox incubator (telemetry data: Chinese authorities, personal communication). The pump system was activated after 5 days in space and injected the fixative solution from the fixative/waste unit into the CC's of FMs. This yielded a final RNA*later* concentration of about 90% (v/v) after mixing. Temperature in CCs was kept at a nominal range of 22 to 24°C before, during, and after fixation (Figure 6). After 17 days in space, the spacecraft was separated from Tjangong-1 and touched ground on November 17, 2011. After landing and recovery of the capsule, samples were retrieved within 6 hours. The ECs were disassembled and stored around 4°C until they arrived in Tübingen on November 25, 2011. In the home laboratory, calli were harvested and stored at −80°C until processing.

### 2.5. Ground Control

Immediately after the launch, the laboratory equipment and cell cultures were brought back to the PITC by Chinese scientists. The ground experiment started with a one-day delay on November 2, 2011 (Figure 5). The EUE was integrated into the Simbox duplicate, according to the position in the flight incubator (experiment/static platform), and kept at 23°C. As in the experiment in orbit, samples were metabolically quenched after 5 days (November 7). The ground experiment ended on November 19. The samples were handled as described for the experiment in orbit.

### 2.6. Experiment Conditions and Specification of Generated Samples

During the Simbox mission, the samples were exposed to different experimental conditions. In the experiment in orbit, FM 16002 was attached to the static platform of the Simbox incubator and experienced 5 days of microgravity (group FS, Flight Static). FM 16001 was centrifuged, resulting in a 1 g control (group FC, in-flight centrifugation). In the ground experiment, the same experimental design was used. FM 16003 was fixed to the static platform (group GS, ground static). In summary, we obtained one biological sample per CC, resulting in two replicates for each FM (front and rear CC) and for each experimental condition, respectively.

### 2.7. Isolation of Total RNA and High-Density Oligonucleotide Arrays

Total RNA was extracted using the RNeasy Plus kit (Qiagen, Hilden, Germany) according to the manufacturer's instructions. Quantity and quality controls were performed and samples were processed using the MessageAmp II-Biotin Enhanced, Single Round aRNA Amplification Kit (Ambion, Life Technologies, Darmstadt, Germany) as described earlier [21, 37]. Fragmented, biotin-labeled aRNA was then submitted to a high throughput microarray analysis (GeneChip Arabidopsis ATH1 Genome Array, Ref: 510690, LOT: 4155830, Affymetrix, Santa Clara, California, USA). Hybridization was performed according to the manufacturer's instructions (for details, see http://www.affymetrix.com/support/technical/manuals.affx). The Affymetrix protocol EukGE-WS2_V4 was used for washing and staining procedures.

### 2.8. Gene Expression Analysis

Expression data were calculated from raw values of the detected signal intensity of hybridization events of all spotted probe sets and saved as  .CEL data files. Microarray data are available in the ArrayExpress database (http://www.ebi.ac.uk/arrayexpress, [38]) under accession number E-MTAB-2518. For integrative data analysis, we used the open-source software Mayday [39]. Normalization was performed using the robust multiarray average method of background-adjustment, quantile-normalization, and median-polish to ensure comparability of arrays and estimate log_2_ expression values [40–42]. Hierarchical clustering was performed by means of the neighbour joining method [43] in order to reconstruct and visualize relationships within expression values due to experiment conditions. The Pearson Correlation coefficient was used to calculate the distance between each experimental condition (FS, FC, and GS) and biological replicates (front and rear CC). The matrix of variant genes was filtered and subjected to a Student's *t*-test (*P* ≤ 0.1) with combined false discovery rate (FDR) correction to identify significantly altered transcripts (*P* < 0.1) between the sample groups FS and FC, FS, and GS, and FC and GS, respectively. Differentially expressed genes were determined by fold change (fc) calculation of log_2_ transformed expression data. Thereby, the threshold was set at −1 ≥ log_2_ (fold change) ≥ 1 for at least 2-fold altered transcripts [40, 41, 44]. Additionally, the Affymetrix probe identifiers were tested by Gene Set Enrichment Analysis (GSEA, [45]) for enrichment of functional ontologies using Gene Ontology terms [46] within Mayday. Thereby, we focused on genes that share their function in identical biological processes for interpreting the genome-wide expression profiles.

## 3. Results

The aim of this experiment was to characterize the transcriptome of* Arabidopsis* semisolid callus cultures after 5 days in space. Due to the availability of an in-flight centrifuge, it was possible to compare expression data with (a) real microgravity samples (thought to yield the microgravity related alterations) and with (b) those from the ground controls (which should deliver effects of nonmicrogravity related spaceflight conditions). This was achieved with high-density oligonucleotide arrays.

### 3.1. Performance of Hardware and Biological Material

**Figure 7:**
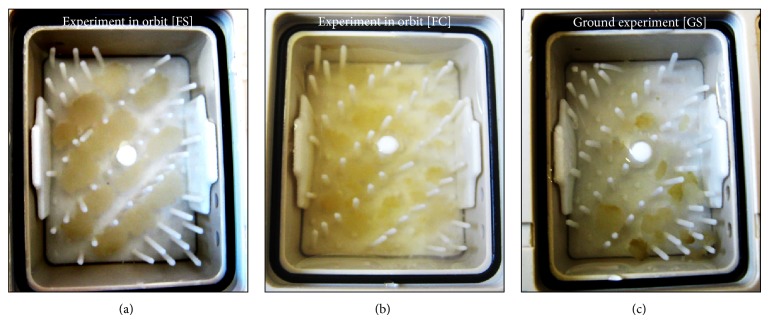
Photograph of* Arabidopsis thaliana* semisolid callus cultures after a 5-day *µ*g cultivation in orbit ((a), FS), 1 g in-flight cultivation ((b), FC) or on ground ((c), GS). The photographs were taken after fixation by RNA*later* and recovery of the spacecraft.

The hardware was thoroughly tested in order to retain viability of the callus cultures for as long as possible. These tests were focused on the biocompatibility of the used materials, gas-exchange properties of membranes, and viability of the cell cultures under the cultivation conditions within the EC. We also recorded the oxygen content within the CC [37]. As this declined from 8 to about 2 mg/L after 5 days, automated sample fixation was set at day 5 after take-off. Mission parameters, such as temperature, were within nominal range during the mission. Radiation measurements recorded increased values. After landing and return of the biological material to the University of Tübingen (Germany), the samples were visually checked. The fixed calli showed good morphology and had well grown during the initial culture of 5 days in space. The calli from the 1 g controls (flight and ground experiment) were smaller compared to those exposed to microgravity (Figure 7).

### 3.2. Biology of Samples and Gene Expression Analysis

**Figure 8:**
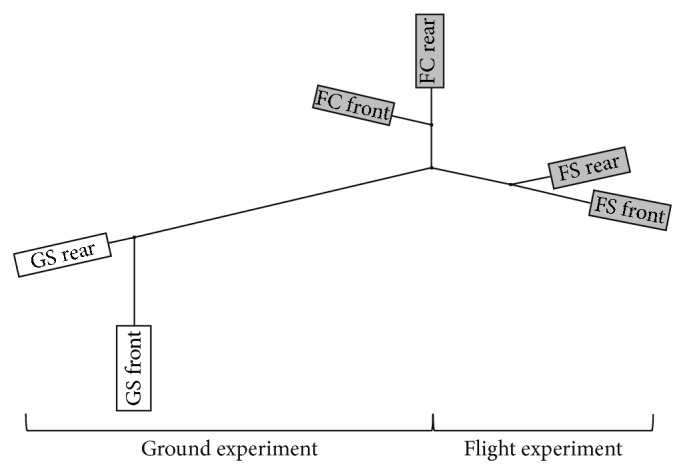
Hierarchical clustering by means of the neighbour joining method of generated sample groups (white: ground experiment; GS: ground static; grey: flight experiment; FS: flight space; FC: in-flight centrifugation). Each EUE consisted of two culture chambers (front and rear chambers, illustrated by boxes).

**Figure 9:**
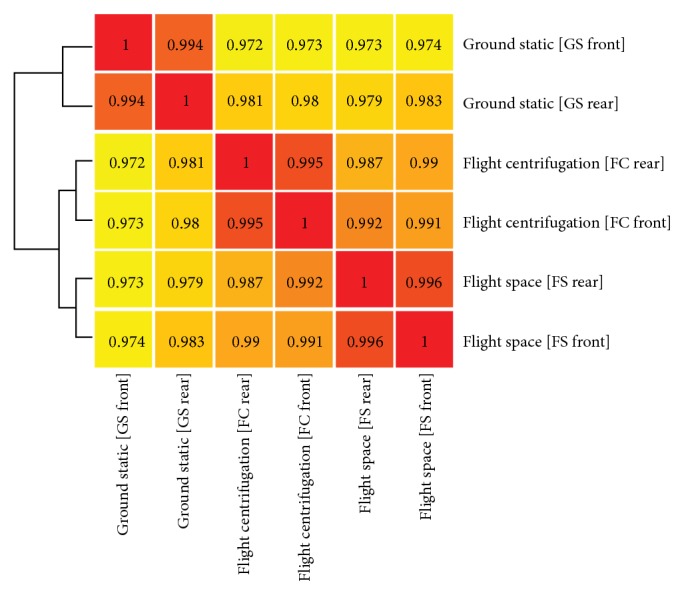
Pearson correlation heat map shows high degree of similarity between front and rear culture chamber of each sample within each sample group. Flight space (FS), in-flight centrifugation (FC), and ground static (GS).

**Figure 10:**
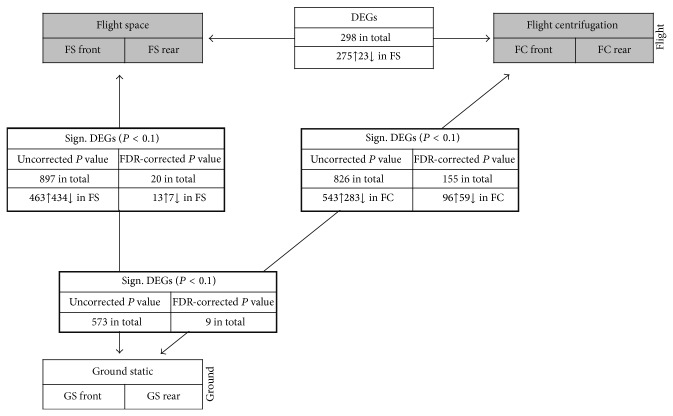
Overview of the number of differentially (fold change (fc) at least 2) and significantly expressed genes (DEGs, *P* < 0.1) within the flight (grey) and ground (white) experiment. The different sample groups are illustrated by boxes. Up- and downregulated transcripts are symbolized by arrows behind the number of altered genes. Genes that are significantly (*P* < 0.1) differentially expressed are shown in boxes framed in black (bold lines).

The quality of the extracted total ribonucleic acid was satisfying for GeneChip hybridization (for RNA quality, see Supplementary Material S2) with clear bands representing the 28S and 18S rRNA. Whole-genome microarray screening was performed for each sample. Due to the limited amount of total RNA, the confirmation of expression data by quantitative real-time PCR was not possible. The data analysis revealed experiment-specific properties of biological replicates which were visualized by hierarchical clustering on the basis of the calculation of the Pearson Correlation coefficient (Figure 8). In this graph, a relatively short distance implies a high correlation between the samples. As obvious from Figure 8, the flight and ground experiment showed group-based clustering. The short distance between FS and FC (FS and FC boxes) in contrast to GS (GS boxes) indicates that nonmicrogravity related spaceflight conditions have major impact. The transcriptome of the biological replicates within the experiment groups (front and rear chamber of FS, FC, GS; *n* = 2) showed a high degree of similarity (Figure 8). This fact was confirmed by heat map generation based on calculated correlations (Figure 9). The Pearson Correlation was about 0.99 between front and rear CC for all three modules (FS, FC, and GS, *n* = 2, Figure 9). Statistical (Student's *t*-test, *P* < 0.1, and FDR correction) and comparative analysis showed a relatively low response of semisolid callus cultures (Figure 10). Interestingly, microgravity conditions did not induce statistically significant changes (*P* < 0.1) at the gene expression level, although 298 genes were at least 2-fold differentially expressed (275 up- and 23 downregulated) within flight space (FS) samples. In contrast, nonmicrogravity related spaceflight conditions interfered with gene expression, considerably. Eight hundred ninety-seven genes were significantly and differentially expressed (at least 2-fold, *P* < 0.1) when 1 g ground and *μ*g exposed flight samples were compared. Among them, 463 were upregulated and 434 genes were downregulated within FS (Figure 10). Comparison between both 1 g controls (in-flight, ground) resulted in 826 significantly (*P* < 0.1) differentially altered genes (543 up and 283 downregulated, Figure 10). Thereby, 573 significant DEGs (*P* < 0.1) were identical in both comparisons (Figure 10).

### 3.3. Identification of Altered Genes after Long-Term Microgravity

For detection of gene expression changes due to *μ*g exposure, we compared data generated out of the sample groups flight space (FS) and in-flight centrifugation (FC). Two hundred seventy-five genes were at least 2-fold differentially upregulated and 23 downregulated (Figure 10). The application of statistics showed that there were no significant (*P* < 0.1) alterations at the expression level after 5 days in space. By means of a Gene Ontology [46] based Gene Set Enrichment Analysis (GSEA), the DEGs were related to common biological processes. In order to identify processes which are specifically influenced by microgravity conditions, we compared overrepresented processes that were identical between sample group FS versus FC and FS compared to GS (Table 1). Most prominent were effects on the translation machinery (Table 1, gene set number 24). Interestingly, all genes that were differentially upregulated and involved in translation processes were chloroplast-encoded. This gene set comprises genes coding for several protein subunits and components of ribosomes (e.g., ATCG00065, ATCG00660, ATCG00770, and ATCG00790) but also the nucleus-encoded translation initiation factor EIF-5A (AT1G13950) that is well known to regulate translation initiation and termination within the cytoplasma of eukaryotes (Table 2). The other part of identified differentially upregulated genes is involved in electron transport chains located within mitochondria (Table 1, gene sets number 4, 8 and 11) such as subunits of the NADH dehydrogenase multi-enzyme complex of the respiratory chain (ATMG00650, ATMG00070, ATMG00580) (Table 2). Mitochondrial electron transport is connected to the production of adenosine triphosphate (ATP). Thus, the gene set representative for ATP biosynthesis was also part of the DEGs (ATCG00120, ATMG00410, ATCG00480, and ATCG00150) (Table 2). Within the 23 downregulated genes (at least 2-fold), no special gene sets could be found, but the largest group codes for heat shock proteins (AT4G27670, AT2G29500, AT5G12020, AT5G59720, AT4G25200, AT1G53540, and AT5G12030).

### 3.4. Attempt to Distinguish between Effects of Microgravity and Nonmicrogravity Related Spaceflight Conditions on Gene Expression

One aim of this investigation was to separate responses to microgravity from those of nonmicrogravity related spaceflight conditions. Until today, only marginal data exist about these effects on plants in space. Thus, we screened for genes that were significantly (*P* < 0.1) altered within spaceflight samples (FS and FC) compared to the 1 g ground control and were identical between FS and FC compared to GS. This overlap yielded 573 significantly altered (*P* < 0.1) DEGs (Figure 10). The GSEA of these genes represented diverse biological processes (Table 1, bold font). The majority of these genes could be related to intracellular signaling pathways such as mitogen-activated protein kinase (MAPK) cascades and protein phosphorylation (Table 1, gene set number 6 and 12). Included were different MAP kinases (e.g., AT1G01560, AT1G73500), serine/threonine/tyrosine kinases (e.g., AT1G20650, AT5G16900, and AT4G38470), and many other kinases (Table 3). Furthermore, we identified genes coding for members of the calcium-binding EF-hand protein family (AT3G01830, AT3G47480) and the WRKY transcription factors 54, 70, and 38 (AT2G40750, AT3G56400, and AT5G22570) that have also transcription regulation activity (Table 3). Additionally, the spaceflight environment other than microgravity had a significant (*P* < 0.1) impact on general stress-responsive (gene set number 20) and defense-related genes (3), especially those involved in the response to oxidative stress and respiratory burst responses (21). These are peroxidases 21, 4, 52, and 25 (AT2G37130, AT1G14540, AT5G05340, and AT2G41480), catalase 3 (AT1G20620), and receptor-like kinases (AT5G46330, AT2G19190). The latter can be induced upon contact with the bacterial protein flagellin which is an important elicitor of the plant defense response. These kinases are also important members of the MAP kinase signaling cascade. Furthermore, general metabolic processes (gene set number 7), protein targeting (13), and rRNA processing (21) were overrepresented due to nonmicrogravity related conditions in space.

## 4. Discussion

The expression data of* Arabidopsis* semisolid callus cultures show alterations in differential gene expression in response to microgravity. However, the influence of the spaceflight environment, in addition to microgravity, is significant.

### 4.1. Identification of Altered Genes after 5 Days of Microgravity

Comparison between microgravity and 1 g space controls revealed about 298 differentially (but not significantly) expressed genes. This number is low in comparison to short-term exposures to microgravity within a range of minutes (TEXUS 47, sounding rocket experiment, [47]) or seconds (14. DLR parabolic flight campaign, [21]). This finding could be due to the small number of biological replicates (2 biological replicates only due to limited material and hardware). However, similar observations are also reported by others. After 4 days in space,* Arabidopsis* plants exhibited only 27 transcripts which were at least 2-fold altered at their expression level [2]. This might indicate that plants respond immediately to a microgravity environment but then adapt to the new situation on the longer run. Also Zhang et al. [32] could also identify only 45 proteins changed in expression after 14 days in space (same mission). Genes with prolonged changes in expression could, however, provide important information about the physiological needs after a few days in space. These include an upregulated group of genes which code for proteins that constitute the ribosomal complex within plastids. These are necessary for translation of mRNA. The upregulation of the mitochondrial electron transport chain could indicate an increased need for ATP. The upregulated expression of NADH dehydrogenase could have the same reason. Interestingly, gene products involved in processes like the response to stress, protein degradation, or programmed cell death appeared not to be altered in expression. The involvement of a series of genes with still unknown functions (not shown) suggests that the space environment induces also unknown cellular processes. Together with the fact that there were no significant changes in gene expression detectable after 5 days of microgravity, lets us suggest that at this stage the impact of a lack of gravitation on cell physiology was not too heavy. The space environment per se, however, causes possibly an increased energy demand, as shown by the upregulation of respiratory components. This aspect should be taken into consideration when plants will be used to provide nutrients, oxygen, and energy on long duration space missions.

Heat shock proteins (HSPs) dominate the group of transcripts which are reduced in amount (not shown). These proteins are involved in many forms of stress response. They enable the folding and membrane translocation of proteins and are thought to reconstitute the tertiary structure of proteins affected by stress events. This way they can increase the stress tolerance. A decreased expression (our study) should thus indicate a lower number of proteins affected in their structure and was also reported for* Arabidopsis in vitro* callus cultures under simulated microgravity conditions (magnetic levitation, magnetic field strength 10.1 Tesla) [48] as well as for the single-cell system of the fern* Ceratopteris richardii* [9]. There are, however, also reports on increased expression of HSPs [15, 16, 21, 24].

A group of plant genes which are always affected by altered gravity are those involved in cell wall modification [2, 49–51]. This reflects the need for increased stability (hypergravity) or more flexibility (microgravity). In the present study, expression of expansins (cell wall loosening) is increased (not shown). This might be the reason for the enhanced size of the microgravity cultures when compared to the 1 g controls (Figure 7).

### 4.2. Impact of the Nonmicrogravity Related Spaceflight Conditions on Gene Expression

The availability of a 1 g reference centrifuge enabled us to screen for genes affected by nonmicrogravity related spaceflight conditions in that we compared expression data between *μ*g exposed and 1 g space with 1 g ground samples. This resulted in a considerable number of identical genes altered in mRNA abundance (573 genes) (Figure 10). We thus assume that this could be due to effects of spaceflight-related environmental conditions, including space radiation. Radiation measurements inside the capsule in a position close to our samples yielded a total dose of 5.9 to 8.1 mSV (milliSieverts) and an equivalent dose of 0.37 to 0.51 mSV/d (data: Chinese authorities). This is considerably more compared to terrestrial conditions (1 to 2 mSV/a) and could be one of the reasons for the alterations at transcript levels, obviously not related to *μ*g. Also Zhang et al. [32] reported a greater difference on protein expression of non-*μ*g conditions. Analysis showed that both experimental conditions (*μ*g and non-*μ*g spaceflight conditions) affect different biological processes (Table 1). Overrepresented processes should not be regarded separately, as they are closely linked together within a plant cell. For example, the formation of reactive oxygen species (ROS) is one of the initial responses upon most kinds of stresses. They are also produced as by-products of redox reactions. They are important second messengers, as well as toxic species, and their cellular levels are closely controlled by detoxification systems [52–54]. The role of ROS in response to environmental changes can, however, also be deduced from alterations in gene products, involved in ROS production and turnover. In this study, we observed that many ROS-related genes are significantly regulated (Table 3). These comprise peroxidases, catalase, and a glutathione S-transferase (Table 3). These proteins are suggested to be part of the stress-induced antioxidant system [55]. Glutathione S-transferases also possess peroxidase activity and can thus prevent cell damage by peroxides, such as hydrogen peroxide [56, 57]. The increase in detoxification-related transcripts appears reasonable, as radiation in orbit consists of highly energetic (HZE) particles from interplanetary galactic sources or results from solar particle events, which could have an impact on cells [58–62]. Wan et al. [63, 64] showed that X-rays, *γ*-rays, protons, and heavy charged particles increased oxidative stress in different cell types, and countermeasures for space radiation effects are the use of antioxidants [62]. Similar responses are probable for plant cells. Therefore, the impact of long-term space radiation on the transcriptome of* Arabidopsis* should be investigated in ground-based studies in simulation testbeds for the space environments [59].

In addition, a range of WRKY transcription factors and components of signaling chains (Ca^2+^-dependent proteins, MAP kinases) were identified (Table 3). These responsive kinases (Table 3) are potentially also modulated by cytosolic fluctuations of H_2_O_2_ and can thus be part of signal transduction chains starting from hydrogen peroxide (for defense-related genes in tomato, see Orozco-Cárdenas et al. [65]). In contrast to other observations to altered gravitation [2, 15], in this study, genes which are defense-, resistance-, and pathogen-related are significantly altered due to non-*μ*g related spaceflight conditions.

## 5. Conclusions

In this study, gene expression changes within* Arabidopsis* wild type semisolid callus cultures were investigated after a 5-day spaceflight and compared to on-board and ground controls. Faced with limited HW capacities (only 3 EUEs) and small amounts of biological material (*n* = 2 for each sample group), high-density oligonucleotide arrays were used to screen for changes at the gene expression level. For future investigations, it would thus be desirable to have flight repetitions and an adequate amount of samples for additional analysis (e.g., qPCR). Unexpectedly, the response of callus cultures to long-term microgravity was less prominent compared to nonmicrogravity related spaceflight conditions. The latter, including space radiation, induced differential and significant expression changes of transcripts that are involved in the stress-induced antioxidant system, signalling chains, and defense-/resistance-related genes. These findings clearly highlight that the use of an in-flight reference centrifuge (1 g in-flight control) should be mandatory during space flight missions.

## Supplementary Material

Supplementary material S1 shows the accelerometer-recorded gravity level profile (x-/y-/z-axis) as measured during the Simbox mission from EZT (Experiment Zero Time) until landing on November 17, 2011 (data: China Manned Space Engineering). Data was provided by Chinese authorities to DLR/Astrium.
Supplementary material S2 shows the formaldehyde agarose gel analysis of extracted RNA from flight (FC) and ground samples (GS), front and rear CC (Culture Chamber).

## Figures and Tables

**Table 1:** Visualization of enriched Gene Ontology categorization terms (GSEA, Gene Set Enrichment Analysis of biological processes). Gene sets identical in FS/FC and FS/GS are not colored; the ones identical in FS/GS, FC/GS and the overlap of both are in bold font (FS = flight space; FC = flight centrifugation, and GS = ground static).

Number	Enriched gene set (biological process)	FS/FC	FS/GS	FC/GS	Overlap
		Gene set size			
1	**ATP catabolic process**	0	**8**	**8**	**7**
2	ATP biosynthetic process	10	9	0	0
3	**Defense response**	0	**20**	**26**	**14**
4	Mitochondrial electron transport chain	7	7	0	0
5	**Lipid metabolic process**	0	**8**	**7**	**6**
6	**MAPK cascade**	0	**29**	**36**	**27**
7	**Metabolic process**	0	**25**	**20**	**16**
8	Mitochondrial electron transport	11	11	0	0
9	**Oxidation-reduction process**	0	**13**	**12**	**8**
10	**Photosynthesis, light harvesting**	0	**5**	**5**	**5**
11	Photosynthetic electron transport chain	5	5	0	0
12	**Protein phosphorylation**	0	**23**	**31**	**18**
13	**Protein targeting to membrane**	0	**12**	**13**	**10**
14	**Regulation of transcription, DNA-dependent**	0	**12**	**11**	**8**
15	**Respiratory burst involved in defense response**	0	**22**	**26**	**21**
16	**Response to chitin**	0	**7**	**6**	**6**
17	**Response to ethylene stimulus**	0	**5**	**6**	**5**
18	**Response to hypoxia**	0	**6**	**9**	**6**
19	**Response to oxidative stress**	0	**15**	**13**	**9**
20	**Response to stress**	0	**9**	**9**	**6**
21	**rRNA processing**	0	**16**	**15**	**14**
22	**Toxin catabolic process**	0	**7**	**7**	**6**
23	**Transition metal ion transport**	0	**10**	**12**	**8**
24	Translation	27	28	0	0
25	**Two-component signal transduction system**	0	**6**	**5**	**5**

**Table 2:** Differentially expressed genes (fold change (fc) at least 2) within the sample group flight space (FS, front/rear CC) compared to in-flight centrifugation (FC). Samples taken after 5-day cultivation at microgravity and sorted according to the overrepresented biological processes identified by GSEA to be the most prominent.

Number	ATG number	Gene name/description	log⁡(fc)	Enriched Gene set (biological process)
1	ATCG00065	Ribosomal protein S12	2.36	Translation
2	ATCG00660	Ribosomal protein L20	2.14	Translation
3	ATCG00770	30S ribosomal protein S8	1.96	Translation
4	ATCG00160	Ribosomal protein S2	1.84	Translation
5	ATCG00790	Ribosomal protein L16	1.8	Translation
6	ATCG00780	Ribosomal protein L14	1.63	Translation
7	AT1G13950	Eukaryotic translation initiation factor 5A-1	1.14	Translation
8	ATCG01120	Ribosomal protein S15	1.11	Translation
9	ATCG00750	Ribosomal protein S11	1.05	Translation
10	ATCG00800	Ribosomal protein S3	1.04	Translation
11	ATMG00650	NADH dehydrogenase subunit 4L	2.3	Mitochondrial electron transport
12	ATMG00060	NADH dehydrogenase subunit 5	1.84	Mitochondrial electron transport
13	AT2G07751	NADH-ubiquinone/plastochinone oxidoreductase	1.75	Mitochondrial electron transport
14	ATCG01050	Subunit of NAD(P)H dehydrogenase complex	1.74	Mitochondrial electron transport
15	ATMG00160	Cytochrome c oxidase subunit 2	1.66	Mitochondrial electron transport
16	ATMG00070	NADH dehydrogenase subunit 9	1.5	Mitochondrial electron transport
17	ATCG00420	NADH dehydrogenase subunit J	1.43	Mitochondrial electron transport
18	ATCG01250	NADH dehydrogenase ND2	1.25	Mitochondrial electron transport
19	ATMG00510	NADH dehydrogenase subunit 7	1.24	Mitochondrial electron transport
20	ATMG00270	NADH dehydrogenase subunit 6	1.24	Mitochondrial electron transport
21	ATMG00580	NADH dehydrogenase subunit 4	1.19	Mitochondrial electron transport
22	ATCG01070	NADH dehydrogenase ND4L	1.13	Mitochondrial electron transport
23	ATCG00120	ATPase *α*-subunit	2.15	ATP biosynthesis
24	ATCG00140	ATPase III subunit	1.59	ATP biosynthesis
25	ATMG00410	ATPase subunit 6	1.56	ATP biosynthesis
26	ATCG00130	ATPase F subunit	1.47	ATP biosynthesis
27	ATCG00480	*β*-Subunit of ATP synthase	1.33	ATP biosynthesis
28	ATCG00150	Subunit of ATPase complex CF0	1.12	ATP biosynthesis

**Table 3:** Differentially (at least 2-fold) and significantly expressed genes (*P* < 0.1, 573 in total) that are identical between flight space (FS) as well as in-flight centrifugation (FC) compared to ground static (GS). Changes are due to nonmicrogravity related spaceflight conditions. The genes are sorted according to the overrepresented biological processes identified by GSEA to be most prominent.

No	ATG number	Gene name/description	log⁡(fc) (*P* value) FS versus GS	log⁡(fc) (*P* value) FC versus GS	Biological process
1	AT1G01560	MAP kinase 11	1.83 (0.034)	2.13 (0.027)	MAPK cascade

2	AT1G73500	MAP kinase 9	1.34 (0.006)	1.37 (0.038)	MAPK cascade

3	AT3G01830	Calcium-binding EF-hand family protein	1.3 (0.032)	1.85 (0.027)	MAPK cascade

4	AT3G47480	Calcium-binding EF-hand family protein	1.24 (0.081)	1.87 (0.037)	MAPK cascade

5	AT2G40750	WRKY DNA-binding transcription factor 54	1.29 (0.008)	1.74 (0.006)	MAPK cascade

6	AT3G56400	WRKY DNA-binding transcription factor 70	1.85 (0.008)	2.19 (0.004)	MAPK cascade

7	AT5G22570	WRKY DNA-binding transcription factor 38	2.52 (0.006)	3.28 (0.004)	MAPK cascade

8	AT3G15500	NAC-domain containing transcription factor 3	2.98 (5.72*E* − 4)	2.63 (0.003)	MAPK cascade

9	AT1G35670	Calcium-dependent calmodulin-independent protein kinase 2	1.2 (0.002)	1.23 (0.003)	Protein phosphorylation

10	AT1G20650	Serine/threonine protein kinase superfamily protein	−1.4 (0.024)	−1.5 (0.018)	Protein phosphorylation

11	AT3G61160	Serine/threonine protein kinase family protein	−1.22 (0.007)	−1.4 (0.008)	Protein phosphorylation

12	AT1G78290	Serine/threonine protein kinase family protein 2C	1.71 (0.019)	2.0 (0.034)	Protein phosphorylation

13	AT4G18640	Serine/threonine protein kinase family protein	1.08 (0.019)	1.07 (0.014)	Protein phosphorylation

14	AT4G18950	Serine/threonine/tyrosine protein kinase family protein	2.53 (0.031)	3.17 (0.02)	Protein phosphorylation

15	AT5G16900	Leucine-rich repeat protein kinase family protein	1.42 (0.023)	2.0 (0.012)	Protein phosphorylation

16	AT1G51890	Leucine-rich repeat protein kinase family protein	2.55 (0.05)	2.64 (0.047)	Protein phosphorylation

17	AT4G11480	Cysteine-rich receptor-like protein kinase family protein	1.56 (0.05)	1.89 (0.033)	Protein phosphorylation

18	AT4G23260	Cysteine-rich receptor-like protein kinase family protein	1.65 (0.068)	2.49 (0.041)	Protein phosphorylation

19	AT4G38470	Tyrosine kinase family protein 46	1.14 (0.008)	1.34 (0.015)	Protein phosphorylation

20	AT1G69790	Protein kinase superfamily protein	1.19 (0.038)	1.12 (0.009)	Protein phosphorylation

21	AT5G53450	Protein kinase	1.88 (0.088)	1.89 (0.075)	Protein phosphorylation

22	AT1G51620	Protein kinase family protein	1.8 (0.052)	2.31 (0.048)	Protein phosphorylation

23	AT3G04530	Phosphoenolpyruvate carboxylase kinase 2	−1.6 (0.06)	−1.19 (0.43)	Protein phosphorylation

24	AT5G63650	Protein kinase 2.5	−1.26 (0.028)	−1.01 (0.032)	Protein phosphorylation

25	AT1G16260	Cell-wall associated protein kinase family protein	1.73 (0.006)	2.13 (0.003)	Protein phosphorylation

26	AT1G68690	Proline-rich extension-like receptor kinase family protein	1.04 (0.002)	1.03 (0.04)	Protein phosphorylation

27	AT5G46330	Flagellin 2-induced receptor-like kinase	−1.85 (0.043)	−2.38 (0.016)	Defense response

28	AT2G19190	Flagellin 22-induced receptor-like kinase	2.48 (0.065)	2.27 (0.075)	Defense response

29	AT2G15120	Disease-resistance family protein	2.68 (0.035)	2.53 (0.04)	Defense response

30	AT1G59780	Disease resistance protein	1.37 (0.092)	1.98 (0.052)	Defense response

31	AT1G63880	Disease resistance protein	−1.81 (0.003)	−1.79 (0.016)	Defense response

32	AT2G39200	Transmembrane domain-containing protein, similar to mildew resistance protein 12	2.6 (0.059)	2.55 (0.063)	Defense response

33	AT1G19610	Pathogenesis-related protein 1.4	−2.17 (0.002)	−2.14 (0.029)	Defense response

34	AT3G20600	Nonrace specific disease resistance protein	1.05 (0.037)	2.12 (0.011)	Defense response

35	AT1G02360	Chitinase family protein	2.6 (0.026)	2.87 (0.019)	Defense response

36	AT3G54420	Chitinase family protein class IV	1.73 (0.055)	2.53 (0.026)	Defense response

37	AT4G21390	Serine/threonine protein kinase family protein	1.5 (0.068)	1.86 (0.031)	Defense response

38	AT3G46280	Protein kinase family protein	1.83 (0.074)	2.3 (0.048)	Defense response

39	AT5G35750	Histidine kinase 2	−1.21 (0.042)	−1.35 (0.026)	Defense response

40	AT2G37130	Peroxidase 21	−3.06 (0.014)	−3.4 (0.008)	Response to oxidative stress

41	AT1G14540	Peroxidase 4	3.35 (0.018)	3.28 (0.02)	Response to oxidative stress

42	AT5G05340	Peroxidase 52	2.15 (0.014)	2.06 (0.019)	Response to oxidative stress

43	AT4G37530	Peroxidase family protein	2.17 (0.035)	2.15 (0.026)	Response to oxidative stress

44	AT2G41480	Peroxidase 25	−1.04 (0.011)	−1.06 (0.034)	Response to oxidative stress

45	AT1G20620	Catalase 3	−1.12 (0.07)	−1.39 (0.052)	Response to oxidative stress

46	AT2G29490	Glutathione S-transferase 19 class tau 1	1.75 (0.07)	1.7 (0.073)	Response to oxidative stress

47	AT3G22370	Oxidase family protein	1.3 (0.013)	1.0 (0.089)	Response to oxidative stress

48	AT4G37220	Stress-responsive protein	2.87 (0.004)	1.87 (0.049)	Response to stress

49	AT4G21870	Heat shock protein 26.5	−1.31 (0.002)	−1.43 (0.012)	Response to stress

50	AT2G38750	Calcium-dependent phospholipid binding protein	1.48 (0.02)	1.15 (0.032)	Response to stress
