# Supplementary material for: A Whole-Genome Microarray Study of Arabidopsis thaliana Semisolid Callus Cultures Exposed to Microgravity and Nonmicrogravity Related Spaceflight Conditions for 5 Days on Board of Shenzhou 8
Source: Biomed Res Int. 2015 Jan 13;2015:547495. doi: 10.1155/2015/547495 (PMC4309294; doi:10.1155/2015/547495)

**Supplementary Material S1:** Accelerometer-recorded gravity level profile (x-/y-/z-axis) as measured during the Simbox mission from EZT until landing on November 17, 2011 (data: China Manned Space Engineering).


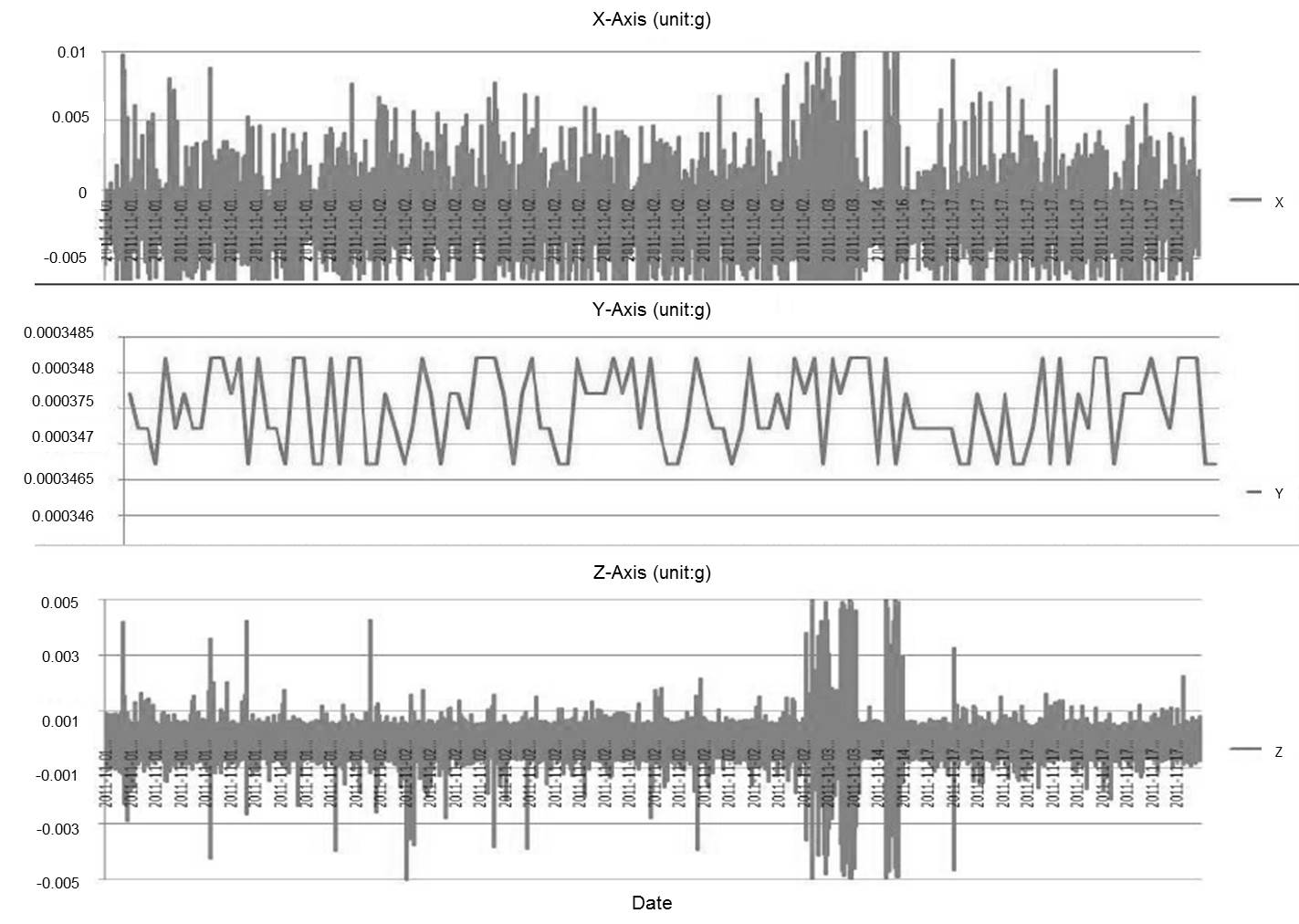


**Supplementary Material S2:** Formaldehyde agarose gel analysis of extracted RNA from flight (FC) and ground samples (GS), front and rear CC (Culture Chamber).


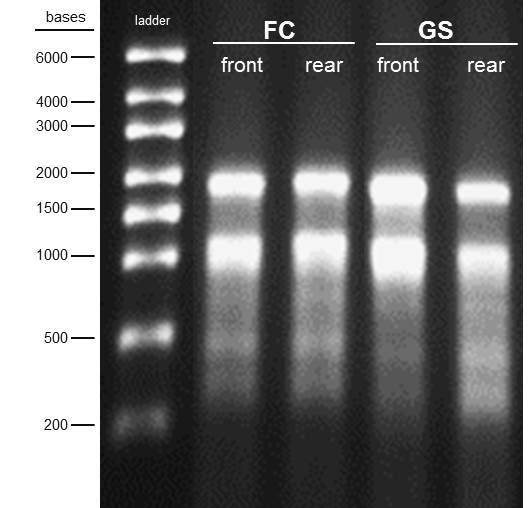

Supplement: Supplementary file 1 — Supplementary material S1 shows the accelerometer-recorded gravity level profile (x-/y-/z-axis) as measured during the Simbox mission from EZT (Experiment Zero Time) until landing on November 17, 2011 (data: China Manned Space Engineering). Data was provided by Chinese authorities to DLR/Astrium. Supplementary material S2 shows the formaldehyde agarose gel analysis of extracted RNA from flight (FC) and ground samples (GS), front and rear CC (Culture Chamber). [file 547495.f1.doc]
